# Predicted effectiveness of vaccines and extended half-life monoclonal antibodies against RSV hospitalizations in children

**DOI:** 10.1038/s41541-022-00550-5

**Published:** 2022-10-27

**Authors:** Zhe Zheng, Daniel M. Weinberger, Virginia E. Pitzer

**Affiliations:** Department of Epidemiology of Microbial Diseases and the Public Health Modeling Unit, Yale School of Public Health, Yale University, New Haven, CT USA

**Keywords:** Viral infection, Epidemiology, Outcomes research, Paediatric research, Respiratory tract diseases

## Abstract

Several vaccines and extended half-life monoclonal antibodies (mAbs) against respiratory syncytial virus (RSV) have shown promise in clinical trials. We used age-structured transmission models to predict the possible impact of various RSV prevention strategies including maternal immunization, live-attenuated vaccines, and long-lasting mAbs. Our results suggest that maternal immunization and long-lasting mAbs are likely to be highly effective in preventing RSV hospitalizations in infants under 6 months of age, averting more than half of RSV hospitalizations in neonates. Live-attenuated vaccines could reduce RSV hospitalizations in vaccinated age groups and are also predicted to have a modest effect in unvaccinated age groups because of disruptions to transmission. Compared to year-round vaccination, a seasonal vaccination program at the country level provides at most a minor advantage regarding efficiency. Our findings highlight the substantial public health impact that upcoming RSV prevention strategies may provide.

## Introduction

Respiratory syncytial virus (RSV) is the leading cause of acute lower respiratory tract infections in children under 5 years of age globally^1^. Currently, no vaccines or antivirals are available for the prevention and treatment of RSV. The sole pharmaceutical prevention strategy is a monoclonal antibody with a short half-life^2,3^. However, the high cost and the short duration of this monoclonal antibody limit its use to high-risk infants in high-income countries^4^. Prevention strategies that benefit the general pediatric population are urgently needed.

Over 40 RSV prophylactic candidates are in pre-clinical or clinical trials^5,6^. Among them, live-attenuated vaccines, long-lasting monoclonal antibodies (mAbs), and maternal vaccines aim to protect pediatric populations^6^. In the current pipeline, RSV vaccines targeting pediatric populations are mainly live-attenuated because of concerns arising from the history of formalin-inactivated RSV vaccine, which caused enhanced disease in seronegative children^7,8^. Phase 3 clinical trials demonstrate that long-lasting mAbs effectively prevent medically attended RSV-associated lower respiratory tract infections (LRTIs) and hospitalizations throughout the RSV season in healthy preterm, late preterm, and term infants^9–11^. At the same time, phase 2b clinical trials of RSV pre-fusion F protein nanoparticle vaccination in pregnant women suggest that maternal immunization can prevent RSV-associated medically significant LRTIs^12–14^. What remains unclear is the expected effectiveness and vaccine impact of different prevention strategies across age groups, especially with different implementation strategies and levels of coverage.

Several studies have evaluated the potential impact of immunization and prevention strategies for RSV in different settings^15–23^. However, the impact of local variations in RSV epidemic dynamics on the predicted effectiveness of seasonal immunization strategies has not yet been investigated. Moreover, little is currently known about the direct and overall effects of different immunization strategies, which may affect the impact of immunization strategies through herd immunity. Incorporating data from various transmission settings and disentangling direct effects from overall effects, our study set out to estimate the potential impact of three RSV prevention strategies that aim to protect pediatric populations and to identify the key factors that affect vaccine impact.

In this study, we assessed the potential impact of live-attenuated vaccines, long-lasting monoclonal antibodies, and maternal vaccines by modifying a previously published transmission dynamic model of RSV and layering on various prevention strategies^24^. The transmission model was validated against state-specific, age-stratified inpatient data from the United States (US) to account for geographical variations in RSV epidemics. We also conducted a sensitivity analysis to identify key drivers of uncertainty that can be informed by future trials and post-implementation studies.

## Results

### RSV hospitalizations before immunization

The transmission dynamic model we developed (see Methods) reproduces the number of monthly RSV hospitalizations and the detailed age distribution of RSV hospitalizations in the four states used for model fitting (New York, New Jersey, Washington, and California) (Supplementary Figs. 1–4). The state-specific fitted parameters capture the notable variations in the observed timing, seasonal amplitude, and age distribution of RSV epidemics across the four states (Supplementary Table 1).

### The overall effectiveness of RSV prevention strategies across age groups over time

**Fig. 1:**
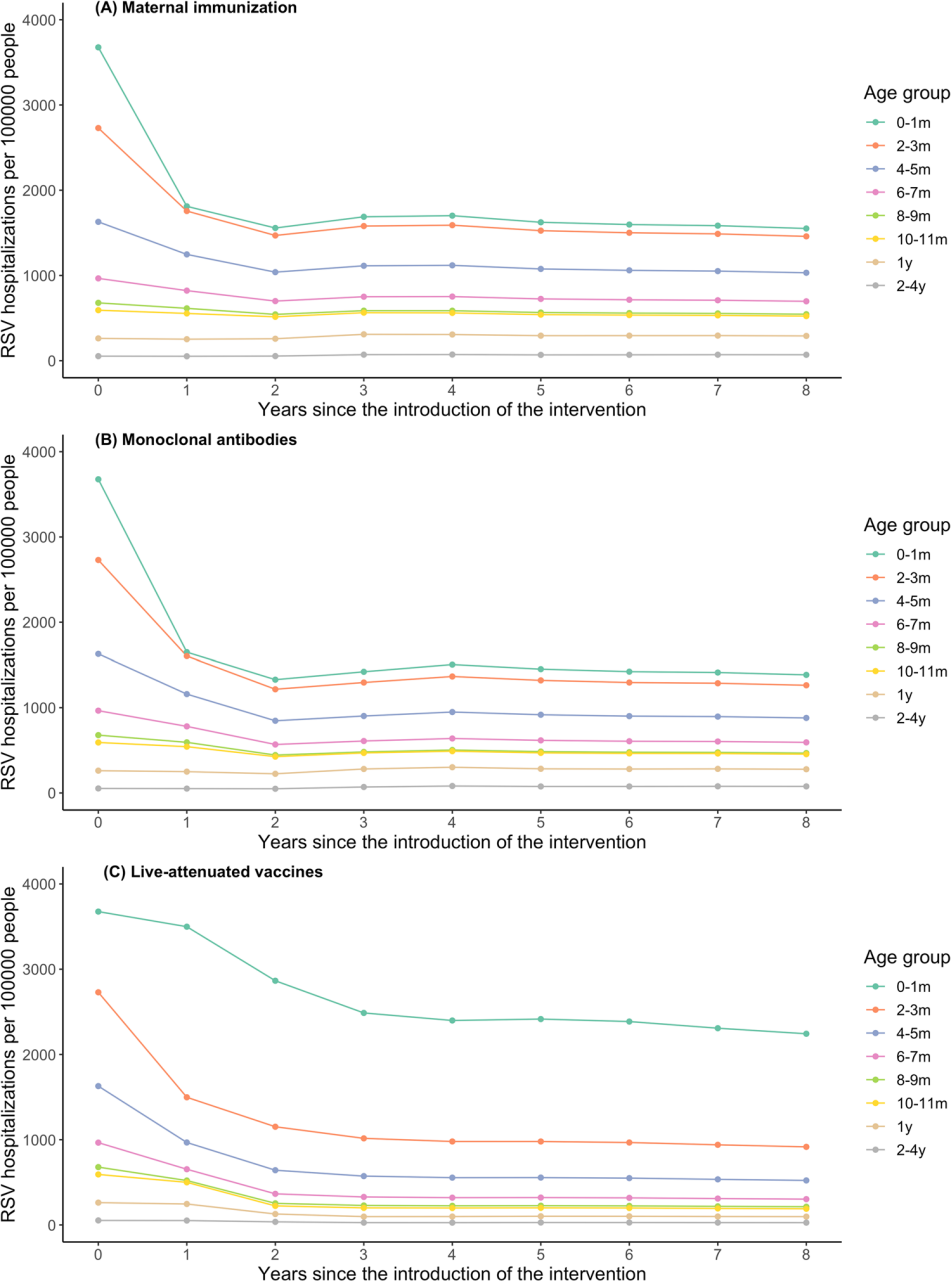
RSV hospitalizations per 100,000 people across age groups before and after the introduction of three RSV prevention strategies. Annual RSV hospitalizations per 100,000 people over time and across age groups are presented for **A** maternal immunization, **B** extended half-life monoclonal antibodies, and **C** live-attenuated vaccines. Year 0 corresponds to the RSV hospitalization incidence before the introduction of RSV prevention strategies. The predicted RSV hospitalization incidence over time is plotted for Years 1 to 8 after the introduction of the intervention. We assumed 85–95% coverage for all three interventions. The color of the lines corresponds to each age group as indicated by the legend.

With high coverage, maternal immunization and long-lasting mAbs are predicted to offer comparable protection against RSV hospitalizations in the most vulnerable population, those under 6 months of age (Table 1). As the duration of transplacentally acquired immunity and vaccine-induced immunity varies between individuals, a small proportion of infants are protected beyond 6 months of age. With realistic coverage, monoclonal antibodies may have a larger overall effect than maternal immunization as the expected uptake of maternal immunization is lower (Table 2). With these two prevention strategies, RSV hospitalizations in children over 1 year of age may slightly increase because the age of the first infection is delayed (Fig. 1A, B and Table 1). Without maternal immunization or mAbs, the average age of first infection is 14.3 months old; the corresponding average age of hospitalization is 8.4 months old (Supplementary Fig. 5). After the introduction of maternal immunization, the average age of first infection is predicted to increase by 5 months (to 19.2 months old); the average age of hospitalization is also delayed by 2 months (to 10.4 months old). A similar effect is predicted following the introduction of long-lasting mAbs (Supplementary Fig. 5).

**Table 1:** Predicted overall vaccine effectiveness.

Vaccination strategy	Maternal immunization (%)	Monoclonal antibodies (%)	Live-attenuated vaccines (%)
0–1 month	53 (34, 63)	58 (31, 76)	31 (11, 45)
2–3 months	40 (25, 49)	50 (25, 64)	64 (44, 79)
4–5 months	28 (17, 36)	41 (19, 54)	65 (50, 77)
6–7 months	19 (11, 25)	32 (14, 45)	66 (53, 77)
8–9 months	11 (6, 16)	24 (9, 37)	66 (54, 76)
10–11 months	4 (0, 8)	16 (3, 28)	65 (54, 76)
1 Yr	−12 (−25, −4)	−11 (−22, −2)	61 (51, 72)
2–4 Yrs	−23 (−56, −7)	−42 (−109, −20)	50 (39, 63)
<5 Yrs	24 (15, 30)	31 (15, 40)	53 (39, 64)

Percentage of RSV hospitalizations averted across age groups in children under 5 years of age in the United States with coverage ranging from 85% to 95%. Medians and 95% prediction intervals are displayed.

Live-attenuated vaccines are predicted to have a strong effect in the target age groups receiving the vaccines, averting about two-third of RSV-associated hospitalizations (Table 1). There is little effect in younger age groups that are not eligible for the vaccine in the first year that vaccination program begins (Fig. 1C). However, a modest effect in the unvaccinated age groups increases over time due to reduced transmission in the population (Fig. 1C). Moreover, compared with the other two strategies, live-attenuated vaccines lead to continued decline in RSV hospitalizations in children over 1 year of age (Table 1 and Fig. 1C).

### Estimated overall and direct effects

**Fig. 2:**
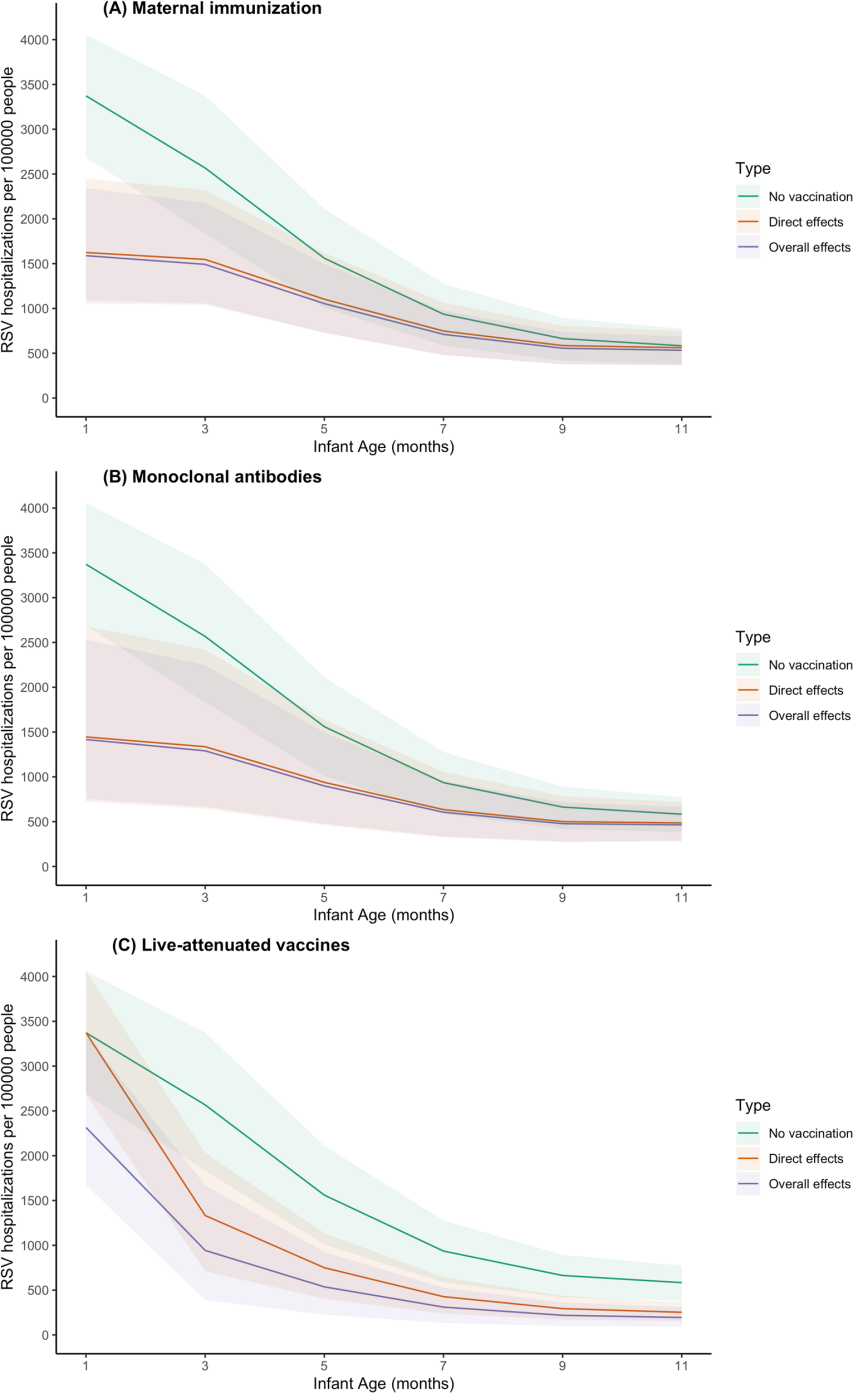
Overall effects and direct effects of three RSV prevention strategies in infants under 1 year of age. The model-predicted RSV hospitalizations per 100,000 people by age is plotted for: **A** maternal immunization, **B** extended half-life monoclonal antibodies, and **C** live-attenuated vaccines. The green lines show the mean RSV hospitalization incidence by age (on the x-axis) assuming no vaccination. The orange lines show the model-predicted RSV hospitalization incidence by age in Year 7 following introduction of the intervention accounting only for the direct effects of RSV prevention strategies (i.e. assuming no reduction in RSV transmission). The purple lines show the model-predicted RSV hospitalization incidence by age in Year 7 accounting for the overall effects of the RSV prevention strategies, which include both the direct and indirect effects among the vaccinated age groups and the indirect effects among the unvaccinated age groups. The color shadows show the 95% prediction intervals for the different scenarios as indicated by the legend.

Maternal immunization and long-lasting mAbs provide direct protection to newborn infants immediately after birth (Fig. 2A, B). The overall effects of maternal immunization and long-lasting mAbs are similar to their direct effects because these strategies are not expected to influence transmission (Fig. 2A). The overall effects of live-attenuated vaccines are greater than the direct effects across all age groups. This difference is especially obvious in infants under 2 months of age who are not eligible to receive vaccines and thus are not protected by direct effects (Fig. 2C).

### Estimated effectiveness under different implementation scenarios

While maternal immunization and long-lasting mAbs provide the greatest protection for neonates, live-attenuated vaccines may provide the largest overall effect for populations under 5 years of age (Tables 1–3). The 95% prediction intervals show the estimates of the predicted effect of the interventions. In the scenario with high year-round coverage, live-attenuated vaccines could avert 208 (95% prediction interval: 153, 273) RSV-associated hospitalizations per 100,000 people, compared with 90 (56, 136) RSV-associated hospitalizations averted by maternal immunization and 120 (68, 165) by long-lasting mAbs.

**Table 2:** Total RSV hospitalizations averted per 100,000 people in children under 5 years of age.

Vaccination Strategy	High year-round coverage	High seasonal coverage	Realistic year-round coverage
Maternal immunization	90 (56, 136)	44 (28, 56)^+^	62 (38, 87)
Monoclonal antibodies	120 (68, 165)	103 (70, 126)^*^	103 (58, 147)
Live-attenuated vaccines	208 (153, 273)	170 (122, 202)^*^	178 (129, 234)

+Vaccination of mothers with due dates between November and March.

*Cover infants at risk (by passive immunization or vaccination) between November and March with a one-time catch-up campaign before the RSV season.

Medians and 95% prediction intervals are displayed.

**Table 3:** Per-dose effectiveness estimates for year-round and seasonal intervention strategies.

Vaccination strategy	Year-round coverage	Nov-mar seasonal coverage	Sep-mar seasonal coverage	Routine+catch-up
Maternal immunization	5.1 (3.2, 7.6)	6.0 (3.8, 7.5)	6.6 (4.2, 8.3)	NA
Monoclonal antibodies	6.8 (3.9, 9.3)	7.2 (3.3, 10.2)	8.1 (4.2, 10.4)	6.6 (4.5, 8.1)
Live-attenuated vaccines	11.8 (8.8, 15.5)	12.1 (8.6, 15.1)	12.6 (9.3, 15.9)	10.0 (7.0, 11.7)

Estimates represent predicted RSV hospitalizations averted per 1000 doses in children under 5 years of age. Medians and 95% prediction intervals are displayed.

Seasonal immunization strategies are predicted to have lower overall effectiveness compared with year-round immunization strategies, which is most evident for the maternal immunization strategy (Table 2). Seasonal maternal immunization is estimated to avert 44 (2856) RSV-associated hospitalizations per 100,000 people, which is half of the hospitalizations averted under the year-round coverage strategy. Seasonal immunization with a one-time catch-up before the RSV season yields a slightly lower overall effectiveness compared with year-round immunization strategies.

Despite the lower impact on rates of hospitalization, seasonal immunization strategies may provide slightly higher per-dose effectiveness (Table 3). For example, under year-round vaccination, maternal immunization is predicted to avert 5.1 (3.2, 7.6) RSV hospitalizations per 1000 doses. Under seasonal maternal vaccination from September to March, the number of RSV hospitalizations averted per 1000 doses is estimated to be 6.6 (4.2, 8.3). The coverage scenario does not affect per-dose effectiveness. While the relative efficiency of seasonal immunization is high for infants under 6 months of age, it is lower for infants aged 6–11 months (Supplementary Table 2).

### Sensitivity analysis

The model parameters that determine the vaccine efficacy are most important in estimating the per-dose effectiveness of each immunization strategy (Supplementary Fig. 6). These include the relative risk reduction of RSV infection in infants born to vaccinated mothers, the duration of protection by monoclonal antibodies, and the immunogenicity of live-attenuated RSV vaccines. The reduced risk of infection in mothers has little impact on the per-dose effectiveness of maternal immunization, even after considering a wider possible range (Supplementary Fig. 6A). For long-lasting mAbs, the duration of transplacentally acquired immunity may also be associated with per-dose effectiveness (Supplementary Fig. 6B). For live-attenuated vaccines, the reporting fraction and transmission parameter are also associated with estimates of per-dose effectiveness (Supplementary Fig. 6C).

## Discussion

As multiple RSV prevention strategies targeting pediatric populations are in the final stages of clinical development and testing, it is important to understand the potential population impacts of these prevention strategies. Applying compartmental models, we set out to predict and compare the overall effectiveness of three different prevention strategies across age groups over time in the United States. Our results suggest that maternal immunization and long-lasting mAbs protect the most vulnerable, those who are under 6 months of age, but they will not provide substantial additional indirect effects for the pediatric population. Live-attenuated vaccines have a lower predicted impact initially, particularly for infants less than 2 months of age, but offer additional benefits by interrupting RSV transmission. In addition, live-attenuated vaccines reduce RSV hospitalizations in children over 1 year of age whereas the other two strategies lead to a slight increase in hospitalization incidence in older children as they delay the first time of infection.

Our study predicts higher overall effectiveness of maternal immunization compared with previous studies^16–19,21^. The main difference comes from the assumptions regarding vaccine efficacy (see Supplementary Table 3, Supplementary Fig. 7, and Supplementary Discussion). Most of the previous studies used efficacy estimates from the phase 3 clinical trial of the Novavax maternal RSV vaccine candidate or assumed a protective duration of 90 days^25^. However, as the clinical trial failed to meet the prespecified success criterion, it is unlikely that a government agency like U.S. Food and Drug Administration will approve this product. Furthermore, a 90-day average protective duration generates a vaccine efficacy estimate below 50% compared to the estimated current level of maternally derived protection. Our study uses updated efficacy estimates based on the latest progression in clinical trials. Our results also suggest that the reduced risk of infection in mothers has little effect in determining the per-dose effectiveness of maternal immunization. The effect of maternal immunization in reducing transmission is predicted to be modest. These results are in line with previous studies^17,26^. Since our model does not consider household structure, the effects of maternal immunization in reducing transmission could be underestimated. However, clinical trials suggested that the risk of RSV disease in vaccinated pregnant women was not different from those who were unvaccinated^25^.

Our results suggest that long-lasting mAbs will have high effectiveness against RSV hospitalizations in infants under 6 months of age. As our models are based on inpatient data of general populations, the high effectiveness indicates the potential of long-lasting mAbs to be administered as universal prophylaxis for every infant, especially if the price is comparable to a vaccine^27^. Our results indicate that the effectiveness of long-lasting mAbs is similar to that of maternal immunization across all age groups. Thus, these two prevention programs are likely to be interchangeable. However, long-lasting mAbs have unique advantages in preventing RSV-associated hospitalizations in preterm infants. Long-lasting mAbs will be a preferable prevention strategy for preterm infants, since preterm labor curtails the total amount of transplacentally acquired antibodies in infants and thus leads to a decreased efficacy of maternal immunization^28^.

Live-attenuated vaccines are estimated to be the most effective immunization strategy in children under 5 years of age (Table 1). Although live-attenuated vaccines do not directly protect the youngest infants, as their immature immune and respiratory system makes them ineligible for receiving live-attenuated RSV vaccines, these vaccines are predicted to provide indirect protection to newborns by decreasing RSV transmission in the entire population. Because an intranasal live-attenuated vaccine can stimulate mucosal immunity, it may reduce RSV infectiousness in vaccine recipients (Supplementary Fig. 8). With the overall reduction in RSV transmission, older adults may also have a lower risk of RSV infection. Therefore, live-attenuated vaccines targeting the pediatric population may also reduce RSV-associated hospitalizations in older adults. While live-attenuated vaccines are predicted to be the best strategy for reducing hospitalization incidence overall among children under 5 years of age, this strategy alone predicts the lowest reduction in RSV hospitalizations among neonates, who are at highest risk of severe outcomes. Combining live-attenuated vaccines targeting infants older than 6 months with either maternal immunization or long-lasting mAbs at birth could potentially provide good protection across the pediatric age spectrum. Future studies are needed to predict the impact and evaluate the cost-effectiveness of such a combined strategy.

One counterintuitive finding of our model predictions for live-attenuated vaccines is that a strategy with one-time catch-up seasonal vaccination is predicted to have the lowest per-dose effectiveness compared with other year-round or seasonal vaccination strategies. This is because with a campaign targeting infants aged 2–9 months, some vaccines will be given to RSV-seropositive infants, who studies suggest may not respond to live-attenuated vaccines^29–32^. Thus, identifying a level of coverage that induces herd immunity while minimizing the wasted doses in seropositive infants and young children could be an interesting topic for future research. If further development of these vaccines leads to increased immunogenicity in seropositive infants and young children, both the overall effectiveness and per-dose effectiveness of live-attenuated vaccines will increase (Supplementary Fig. 9 and Supplementary Table 4).

In contrast to an earlier finding that suggested a seasonal RSV vaccination strategy was much more efficient than a year-round vaccination strategy^20^, our estimates of per-dose effectiveness of the seasonal vaccination strategy are only slightly higher than the year-round vaccination strategy. One major difference between our study and the previous study is that we estimate the per-dose effectiveness in children under 5 years of age, while they looked at infants under 6 months old. We find that although seasonal vaccination is predicted to be more efficient in infants under 6 months of old, infants aged 6–11 months are not well protected by a seasonal vaccination strategy (Supplementary Table 2). This is because infants born in late spring and summer, who will not be protected by a seasonal immunization strategy, will encounter their first RSV season when they are over 6 months of age. Furthermore, our study considers state-specific RSV seasonality within the US, while the previous study was done at the national level across low- and middle-income countries. With high spatial variations in RSV timing in the US^24,33^, a country-level seasonal vaccination strategy is unlikely to be efficient^34^.

Our study is subject to several limitations. First, the overall effectiveness estimates of our study are highly dependent on the input efficacy. Although we use the latest efficacy estimates from clinical trials and apply a reasonable lower bound as input parameters, efficacy estimates may change as clinical trials progress. Therefore, our overall effectiveness estimates need to be interpreted with caution. These analyses are not intended to provide a precise point estimate for each strategy. Instead, the focus is on comparison between the three prevention strategies, particularly with respect to the overall effectiveness in different age groups. Second, the estimates of RSV-associated hospitalizations only reflect a proportion of the “true” disease burden. This limitation will not affect the overall effectiveness, since it is measured as a percentage decrease. However, underreported RSV-associated hospitalizations will lead to underestimates of per-dose effectiveness. This may explain why estimates of RSV-associated hospitalizations averted per 1000 infants immunized from clinical trials with active surveillance are twice what we estimate in our study^11^. Third, we do not consider household structure in our analysis, which may lead to underestimated effects of maternal immunization in reducing transmission. Nonetheless, a previous study that considered household structure also suggested that maternal immunization would induce little herd immunity^17^. Fourth, live-attenuated vaccines may have protective effects in seropositive children that we do not account for in our analysis. We assume that live-attenuated vaccines have no effect on seropositive children because phase I clinical trial data suggested a comparable antibody level in seropositive children before and after vaccination. However, antibody level may not directly translate into vaccine protection. If live-attenuated vaccines confer additional protection among seropositive children, we anticipate a higher overall effectiveness and per-dose effectiveness. On the contrary, if live-attenuated vaccines cannot confer protection among infants aged 2–3 months because of their immature immune system, we anticipate a lower overall and per-dose effectiveness. Lastly, our study does not include a southern state such as Florida or Texas, which have non-canonical RSV seasonality. Nonetheless, as southern states have more year-round RSV circulation, we anticipate the seasonal immunization strategy will have even less of an efficiency advantage compared with a year-round strategy in these states.

In conclusion, we use compartmental models within a Bayesian framework to estimate state-specific transmission parameters and incorporate the uncertainties in transmission settings to predict the overall effectiveness of three RSV prevention strategies in detailed age groups over time. We predict that maternal immunization and long-lasting mAbs will be highly effective in infants under 6 months of age, while a live-attenuated vaccine will be the most effective immunization strategy for children under 5 years of age as it is predicted to provide both direct and substantial indirect protection. A seasonal vaccination program at the country level provides only a slight efficiency advantage over a year-round vaccination program. Our findings join previous research highlighting the substantial public health impact that upcoming RSV prevention strategies may provide.

## Methods

### Data sources

Individual-level hospital discharge data were obtained from the State Inpatient Databases of the Healthcare Cost and Utilization Project, maintained by the Agency for Healthcare Research and Quality (purchased through the HCUP Central Distributor)^35^. The data covered July 2005 to June 2014 for three states (New York, New Jersey, and Washington) and from July 2003 to June 2011 for California. Variables included age at admission (in months for children under 5 years of age and in years otherwise), International Classification of Diseases Ninth Revision (ICD-9) coded diagnoses (multiple fields), and the calendar month and year of hospital admission. Hospitalization was defined as due to RSV if any of the discharge diagnostic codes included 079.6 (RSV), 466.11 (bronchiolitis due to RSV), or 480.1 (pneumonia due to RSV), based on ICD-9. We initialized the transmission dynamic model in 1981 using a total population size equal to the population in the four states in 1980, which was obtained from the 1981 U.S Census Report^36^. We assumed the population age structure of each state remained stable over time and was equal to the age structure for 2010^37^. We stratified the population into 13 age categories: infants younger than 2 months, 2–3 months, 4–5 months, 6–7 months, 8–9 months, 10–11 months, 1 year, 2–4 years, 5–9 years, 10–19 years, 20–39 years, 40–59 years, and ≥60 years. Birth rate by year and state was obtained from the Centers for Disease Control and Prevention vital statistics^38^. We assumed individuals aged into the next age group exponentially, with the rate equal to the inverse of the length of the age class. We adjusted the net rate of immigration/emigration and death to produce a rate of population growth similar to the observed growth.

### Transmission dynamic models

**Fig. 3:**
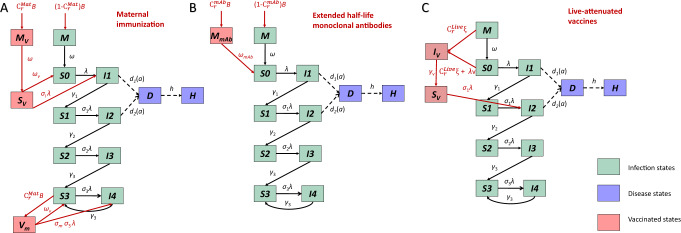
Transmission model structure and immunization strategies. **A** the maternal immunization strategy, **B** extended half-life monoclonal antibodies, and **C** live-attenuated vaccines. The green compartments represent RSV transmission dynamics. Compartments *M* stand for infants who are protected by maternally derived immunity. Compartments *S* stand for susceptible status. The subscripts of *S* compartments represent the number of previous infections. Compartments *I* stand for the infected and infectious status. The subscripts of *I* compartments indicate whether current infection is the first, second, third or subsequent infections. The purple compartments are the observational-level disease states, where *D* stands for lower respiratory tract diseases and *H* stands for hospitalized. The pink compartments are immunized states. In panel **A**, $M_v$ represents infants who are born to vaccinated mothers and are fully protected by maternally derived immunity. After the transplacentally acquired immunity wanes, infants who are born to vaccinated mothers become susceptible ($S_v$) to RSV infection with a reduced risk. $V_m$ represents the vaccinated mothers. In panel **B**, $M_{mAb}$ represents infants who receive long-lasting mAbs at birth. In panel **C**, $I_v$ represents the newly vaccinated infants who can shed vaccine viruses. After the acute vaccine-shedding period, vaccinated infants gain partial immunity and become less susceptible to RSV infection ($S_v$), comparable to the $S_1$ state.

We extended a previously published age-stratified RSV transmission model^24,39^. This model assumes newborn infants are protected against RSV infections because they acquire neutralizing antibodies transplacentally from their mothers and/or have few contacts outside the household. With time, transplacentally acquired antibodies and cocooning effects wane, and infants become susceptible to infection. Following each infection, individuals gain partial immunity that lowers both their susceptibility to subsequent infections and the duration and infectiousness of subsequent infections (see Fig. 3). The risk of lower respiratory disease depends on both the number of previous infections and age at infection in the model^24,39^. We assumed frequency-dependent age-specific contact patterns, which were obtained from previous studies that projected contact patterns for the United States^40^. Since RSV epidemics are highly seasonal and vary across states^24^, we included state-specific seasonality terms that account for the amplitude and timing of seasonal variation in the transmission rate of RSV.

We defined a seasonal age-specific force of infection that varies with time. The force of infection $\lambda _a\left( t \right)$ for age group *a* and time *t* is defined as:

1 $$\lambda _a\left( t \right) = \left( {1 + b_1{{{\mathrm{cos}}}}\left( {\frac{{2\pi t - \phi }}{{12}}} \right)} \right)\mathop {\sum}\nolimits_k {\beta _{a,k}} \left( {I_{1,k}\left( t \right) + \rho _1I_{2,k}\left( t \right) + \rho _2I_{3,k}\left( t \right) + \rho _2I_{4,k}\left( t \right)} \right)/N_k\left( t \right)$$

It contains three major components: the seasonal transmissibility of RSV, the age-specific transmission parameter, and the transmissibility related to the number of infections. The seasonal dynamic of RSV is represented by $\left( {1 + b_1{{{\mathrm{cos}}}}\left( {\frac{{2\pi t - \phi }}{{12}}} \right)} \right)$, where $b_1$ is the amplitude of seasonality in transmission and *ϕ* is the timing of peak transmissibility. The transmission parameter $\beta _{a,k}$ is the product of the per capita probability of transmission given contact between an infectious and a susceptible individual (*q*) and contact rate between age group *k* and age group $a$ ($C_{a,k}$). The age-specific contact patterns were obtained from previous studies that projected the contact patterns to the United States^41,42^. These two components are multiplied by the number of infectious individuals of age *k* who have been infected one, two, three and four or more times at time *t*: $\left( {I_{1,k}\left( t \right) + \rho _1I_{2,k}\left( t \right) + \rho _2I_{3,k}\left( t \right) + \rho _2I_{4,k}\left( t \right)} \right)/N_k\left( t \right)$, where the relative infectiousness of second and subsequent infections are denoted as $\rho _1$ and $\rho _2$, respectively; the total population of age *k* at time *t* is denoted as $N_k\left( t \right)$. We stratified the population into 13 age groups considering their risk of developing severe RSV disease and contact patterns.

The transmission dynamic process is linked to observed inpatient data. We assume that every infected individual has a probability $h_{i,a}$ of developing severe RSV disease that requires hospitalization, which depends on infection order *i* and age *a*, and a fraction *θ* of RSV hospitalizations will be recorded in the inpatient datasets:

2 $$\begin{array}{l}H_a\left( t \right) = \theta \ast \left( \lambda _a\left( t \right)\left( S_{0,a}\left( t \right)h_{p,a} + \sigma _1S_{1,a}\left( t \right)h_{s,a}\right.\right.\\ \qquad\qquad\left.\left. +\, \sigma _2S_{2,a}\left( t \right)h_{t,a} + \sigma _3S_{3,a}\left( t \right)h_{t,a} \right) \right)\end{array}$$

where $H_a\left( t \right)$ is the number of RSV hospitalizations in age group *a* at time *t* and $\lambda _a\left( t \right)$ is the force of infection that age group *a* experiences at time $t$. The fully susceptible individuals of age *a* who have never been infected before are denoted as $S_{0,a}\left( t \right)$. The number of susceptible individuals of age *a* who have been infected one, two, and more times at time $t$ are denoted as $S_{1,a}\left( t \right),S_{2,a}\left( t \right),$ and $S_{3,a}\left( t \right)$, respectively; $\sigma _1,\sigma _2,$ and $\sigma _3$ represent the reduced susceptibility to RSV infection following the first, second, and more infections due to the partial immunity gained after each infection. $h_{p,a}$, $h_{s,a}$ and $h_{t,a}$ are the proportion of the first, second, and subsequent infections in age group $a$ that require hospitalizations, respectively.

The majority of the parameters used in the transmission models were fixed based on data from cohort studies conducted in the US and Kenya (Table 4). We estimated a few key parameters that could potentially affect vaccine effectiveness by fitting transmission dynamic models to the hospitalization data from New York, New Jersey, Washington, and California. The estimated parameters included the per capita probability of transmission given contact between an infectious and susceptible individual, the amplitude of seasonality, the seasonal offset, the waning rate of transplacentally acquired immunity, and the reporting fraction (i.e., the probability that a hospitalization caused by RSV is coded as such in the patient record).

**Table 4:** Model parameters.

Parameter description	Symbol	Parameter value or prior value	Reference	Note
*Transmission dynamic models*				
Duration of infectiousness				
First infection	$1/\gamma _1$	10 days	^65–67^	
Second infection	$1/\gamma _2$	7 days		
Subsequent infection	$1/\gamma _3$	5 days		
Relative risk of infection following				
First infection	$\sigma _1$	0.76	^58–61^	
Second infection	$\sigma _2$	0.6		
Subsequent infection	$\sigma _3$	0.4		
Relative infectiousness				
Second infections	$\rho _1$	0.75	^58,59,68^	
Subsequent infections	$\rho _2$	0.51		
Proportion of RSV infections leading to hospitalization				
First infection			^68–74^	The probability of hospitalization given infection was estimated as the product of the probability of hospitalization given lower respiratory tract infections, the probability of lower respiratory tract infections given symptomatic infection (*I*_*S*_), and the probability of symptoms given infection: $\Pr \left( {hosp{{{\mathrm{|}}}}I} \right) = \Pr \left( {hosp{{{\mathrm{|}}}}LRI} \right) {\times} \Pr \left( {LRI{{{\mathrm{|}}}}I_s} \right) {\times} \Pr \left( {I_s{{{\mathrm{|}}}}I} \right)$ We estimated the age-specific probability by fitting polynomial regressions to reported aggregate probabilities for 3-month or 6-month age groups
0–1 months old	$h_{p,0 - 1}$	0.082		
2–3 months old	$h_{p,2 - 3}$	0.048		
4–5 months old	$h_{p,4 - 5}$	0.027		
6–7 months old	$h_{p,6 - 7}$	0.016		
8–9 months old	$h_{p,8 - 9}$	0.012		
10–11 months old	$h_{p,10 - 11}$	0.012		
1 year old	$h_{p,1y}$	0.010		
2–4 years old	$h_{p,2 - 4y}$	0.007		
$\ge 5$ years old	$h_{p,5 + y}$	0.001		
Second infection	$h_{s,a}$	0.4*$h_{p,a}$	^59^	
Third+ infection	$h_{t,a}$	$0$	^75^	
Waning rate of maternal immunity and cocooning effects (1/months)	$\omega$	$logN( - 1,0.6)$	^46–49,76^	
Transmission parameter*	$q$	$N(3,1)$	^39^	Truncated at (1, 5)
Amplitude of seasonality	$\alpha$	$N(0.2,0.05)$	^39^	
Timing of seasonality	$\phi$	$N(3.4,1)$	^39^	Truncated at (0, 2$\pi$)
Reporting fraction	$\theta$	$beta(2,2)$	^39^	
*Vaccine efficacy parameters*				
Relative risk reduction in infants	$\sigma _i$	84.7% (50.0%–97.6%)	^5,12,45^	Maternal immunization
Relative risk reduction in mothers	$\sigma _m$	90.0% (80.0%–100.0%)	^12,25,45^	Maternal immunization
The duration of vaccine-induced protection	$1/\omega _v$	150 days	^12,46–49^	Maternal immunization
The duration of protection of extended half-life monoclonal antibodies	$1/\omega _{mAb}$	275 days (150 days – 400 days)	^9,27,47^	Extended half-life monoclonal antibodies
The probability of seroconversion	${\upxi}$	90.0% (80.0%–100.0%)	^39,50^	Live-attenuated vaccines
Duration of shedding of vaccine virus	$1/\gamma _v$	5 days	^39,50^	Live-attenuated vaccines
*Coverage parameters*				
Ideal coverage	${{{\mathrm{C}}}}_i$	85.0%–95.0%		
Realistic coverage				
Maternal immunization	${{{\mathrm{C}}}}_r^{Mat}$	53%–70%	^50^	
Extended half-life monoclonal antibodies	${{{\mathrm{C}}}}_r^{mAb}$	70%–82%	^52^	
Live-attenuated vaccines	${{{\mathrm{C}}}}_r^{Live}$	70%–86%	^52^	

*The basic reproductive number (*R*_0_) was estimated from $R_0 = \frac{{{{{\mathrm{det}}}}(\beta _{a,k})}}{{\gamma _1}} = \frac{{{{{\mathrm{det}}}}(qC_{a,k})}}{{\gamma _1}},$ using the next-generation matrix method; the transmission parameter *q* was fitted to the data, $C_{a,k}$ is the contact matrix scaled by the proportion of the population within each age class.

The model fitting process had two steps. We initially seeded the transmission dynamic model with one infectious individual in each age group except for infants under 6 months in July 1981 and used a burn-in period of 24 years in New York, New Jersey, Washington, and 22 years in California to reach quasi-equilibrium. We first used maximum likelihood to fit the model to the observed number of hospitalizations for each state. We then used the maximum likelihood estimates of the number of individuals in each infection state in each age group in July 2005 or July 2003 to initialize the model and refit the transmission dynamic model using Bayesian inference with a gradient-based Markov chain Monte Carlo (MCMC) algorithm. The Bayesian inference allowed us to explore the full parameter space, while the outputs from the maximum likelihood estimation provided a reasonable starting point. The likelihood was calculated by assuming the number of hospitalizations in the entire population in each calendar month was Poisson-distributed with a mean equal to the model-predicted number of hospitalizations, and that the observed age distribution was multinomial-distributed with probabilities equal to the model-predicted distribution of RSV hospitalizations in each age group in children under 5 years in both fitting processes. The prior distribution for each parameter was set as weakly informative (Table 4 and Supplementary Fig. 11). For each state, we sampled 2000 times from 4 chains from the joint posterior distribution of model parameters using STAN^43^ (a gradient-based sampling technique) in R version 4.0.2. We assessed convergence with R-hat, pair plots, and trace plots (Supplementary Fig. 12)^44^.

### Modeling F-protein-based vaccines for pregnant women

We assumed successful maternal vaccination increases the level of transplacentally acquired immunity among infants born to vaccinated mothers and thus reduces the risk of infection in infants after the natural transplacentally acquired immunity wanes. The lower bound of the relative risk reduction was set at 50% because vaccine candidates are unlikely to be approved if the vaccine efficacy is lower than 50%^5,45^. The mean and upper bound of relative risk reduction was based on the vaccine efficacy estimates from the exploratory analysis of Phase 2b Pfizer RSVpreF maternal vaccine, which suggested an 84.7% (95% CI 21.6–97.6%) vaccine efficacy against medically attended RSV lower respiratory tract illness in infants up to 6 months of age. The duration of vaccine-induced protection was assumed to be 5 months (150 days) because the natural transplacentally acquired immunity was estimated to last for approximately one month^46–49^. We assumed the vaccine may provide additional immunity to pregnant women beyond the natural immunity they already acquired, reducing their risk of getting RSV infection and further protecting their neonates and children through disruptions to transmission. The realistic coverage of maternal immunization was informed by the coverage of maternal influenza vaccination (53–70%)^50^. To test whether model structure affects effectiveness estimates, we did a sensitivity analysis using a model structure similar to the model structure used for the extended half-life mAbs (see Supplementary Fig. 7). We also tested a shorter duration of vaccine-induced protection that lasted for 90 days and a lower risk of infection in vaccinated mothers to see how it affects the estimates of effectiveness (Supplementary Table 3).

### Modeling extended half-life monoclonal antibodies for newborns

We assumed that after the successful administration of extended half-life monoclonal antibodies at birth, immunized infants will have prolonged immunity against RSV infection. Efficacy is determined by the waning rate of prophylaxis, ω_mab_; we assumed that the average duration of protection (1/ω_mab_) was 275 days (95% CI 150–400 days)^9,27,51^. After this prolonged immunity against RSV wanes, immunized infants will become susceptible to RSV infection and will have the same risk of infection as unimmunized infants. The realistic coverage of extended half-life monoclonal antibodies at birth was assumed to be the same as the coverage of hepatitis B vaccine birth dose (70–82%)^52^.

### Modeling live-attenuated vaccines for seronegative infants

We assumed live-attenuated vaccines will target infants 2–3 months of age with a single dose, as suggested in the pediatric vaccine development plan from GlaxoSmithKline and WHO Preferred Product Characteristics for Respiratory Syncytial Virus (RSV) Vaccines^53,54^. We assumed a successful live-attenuated vaccination (with seroconversion probability ξ set at 0.9 (95% CI 0.8–1)) is comparable to natural infection, since live-attenuated vaccines can induce both humoral and cellular immune responses^55^. After the administration, vaccinated infants can shed vaccine viruses and “infect” susceptible seronegative infants with the vaccine strain, thereby conferring contact immunity^56,57^. After the acute vaccine-shedding period, vaccinated infants gain partial immunity and become less susceptible to RSV infection, comparable to immunity from one natural infection (i.e. a relative risk of infection of 0.76 and a relative risk of hospitalization given infection of 0.4 compared to unvaccinated infants, based on data from birth cohort studies^58–61^). The realistic expected coverage of live-attenuated RSV vaccines was informed by the coverage of rotavirus vaccine (70%–86%)^52^.

To evaluate the indirect effect resulting from vaccine shedding, we tested a scenario of live-attenuated vaccine without vaccine shedding as a sensitivity analysis (see Supplementary Fig. 8). As there is potential for a booster dose of live-attenuated vaccine in infants, we also tested a delivery strategy that included one booster dose for infants aged 4–5 months as a sensitivity analysis (see Supplementary Fig. 9). We assumed a booster dose would also induce both humoral and cellular immune responses comparable to an additional natural infection.

### Effectiveness calculations

We simulated the impact of each intervention strategy for 8 years following implementation. To incorporate uncertainty in the transmission model parameters, we combined the posterior distributions of the model parameters from all four US states. We used Latin hypercube sampling to jointly sample 1000 times from the uncertainty distributions for the transmission model parameters and the efficacy parameters of each prevention strategy; the 2.5 and 97.5 percentiles of the resulting simulations were used to generate 95% prediction intervals. To directly compare the different prevention strategies, we also considered a “high coverage” scenario in which coverage was sampled uniformly from the range of 85–95%.

The overall effectiveness of prevention strategies against RSV was measured as the percentage reduction in RSV hospitalizations after implementation compared to the predicted number of RSV hospitalizations with no prevention strategies. We also compared the RSV hospitalizations per 100,000 people in each age group after vaccine introduction for 6 years (in Year 7) with high coverage to the no prevention scenario. By definition, the direct effect of an intervention is the difference in disease incidence between vaccinated and unvaccinated individuals when all other quantities (in particular, the transmission rate in the population) are comparable^62,63^. Therefore, to calculate the expected RSV attack rate attributable to the direct effect of vaccination only, we estimated the RSV hospitalizations per 100,000 people when holding force of infection (i.e. the per-susceptible transmission rate) at the same level as in an unvaccinated population. The indirect effect can then be estimated as the difference between the overall effect and the direct effect. To measure per-dose efficiency, we divided the overall effectiveness by the total doses given in each prevention strategy.

We compared year-round and seasonal vaccination strategies. The seasonal vaccination strategy was informed by current national recommendations for RSV prophylaxis in the United States, which starts on November 1 and lasts for 5 months for most states. For maternal immunization, we assumed seasonal vaccination targeting mothers with expected due dates during RSV season (November 1 to March 31). For long-lasting monoclonal antibodies, we assumed infants under 6 months who are born outside of the RSV season will receive the mAbs one time on November 1, right before RSV season; infants who are born between November 1 and March 31 will receive the mAbs at birth. For live-attenuated vaccines, we assumed infants aged 2–9 months will be vaccinated on October 1 so that they reach peak antibody titers on November 1; younger infants will be immunized at 2 months of age between October 1 and the end of February so they will be protected from November to the end of March. We also evaluated alternative seasonal vaccination strategies that immunize newborns or infants at 2 months of age (1) between November and March or (2) between September and March.

To determine the efficiency of seasonal prevention strategies compared to year-round prevention, we calculated the ratio of per-dose effectiveness between a seasonal program and a year-round program in each age group. That is,

3 $$\frac{{\left( {H_{k,no\,vacc} - H_{k,seasonal}} \right){{{\mathrm{/}}}}V_{seasonal}}}{{\left( {H_{k,no\,vacc} - H_{k,year - round}} \right){{{\mathrm{/}}}}V_{year - round}}}$$

where $(H_{k,no\,vacc} - H_{k,seasonal})$ and $(H_{k,no\,vacc} - H_{k,year - round})$ are the number of RSV hospitalizations averted in age group *k* in a seasonal and year-round vaccination program, respectively, and $V_{seasonal}$ and $V_{year - round}$ are the number of vaccine doses given in a seasonal and year-round vaccination program, respectively.

### Variable importance

We used random forest analysis to assess variable importance, since it performs better with non-linear and correlated parameters compared to traditional approaches. Variable importance was measured by the conditional permutation importance method, which measures the prediction error on the out-of-sample portion of the data before and after permuting each predictor variable. This method produces less bias when predictors are correlated compared with the node impurity method or any unconditional permutation importance measure^64^. We explored a wide uncertainty of coverage 40–95% to evaluate the relative importance of coverage in each prevention strategy, along with the uncertainty in the other model parameters. The relative importance was rescaled to be between 0 and 100 to make it comparable across the three prevention strategies.

### Reporting summary

Further information on research design is available in the Nature Research Reporting Summary linked to this article.

## Supplementary information

supplementary document

REPORTING SUMMARY

## Data Availability

The hospitalization data are not available publicly but can be obtained upon signing a data use agreement with the Agency for Healthcare Research and Quality.

## Code availability

The code that supports the findings of this study are available via GitHub (https://github.com/weinbergerlab/rsv-transmission-model).
