# Supplementary material for: Predicted effectiveness of vaccines and extended half-life monoclonal antibodies against RSV hospitalizations in children
Source: NPJ Vaccines. 2022 Oct 27;7:127. doi: 10.1038/s41541-022-00550-5 (PMC9612629; doi:10.1038/s41541-022-00550-5)
Supplement: Supplementary file 1 — supplementary document [file 41541_2022_550_MOESM1_ESM.pdf]

Supplementary results

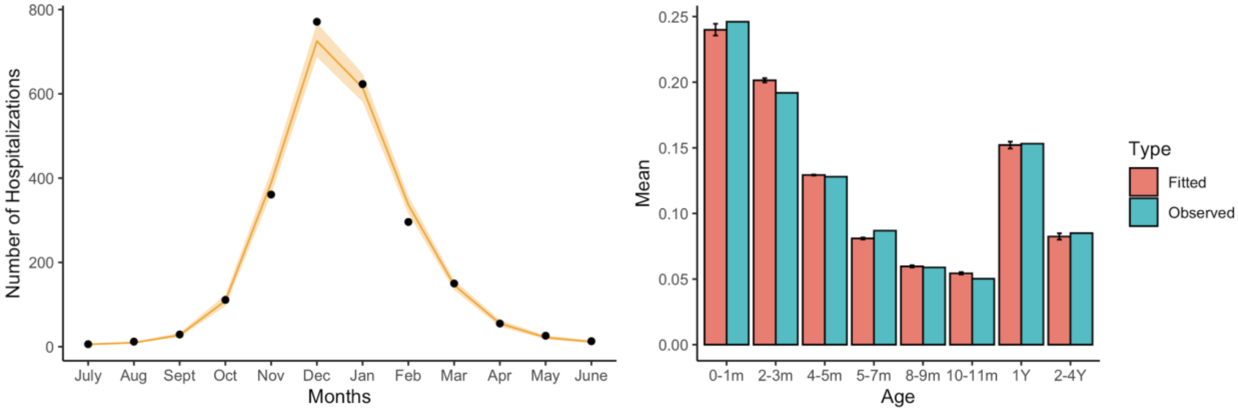

Supplementary Figure 1. Model fit to mean number of RSV hospitalization per month and age distribution in New Jersey. On the left panel, the ICD9-CM coded hospitalization data is represented by the dots. The median of the fitted model is shown in solid yellow line while the shaded area around the line shows the 95% credible interval. On the right panel, the proportion of hospitalizations in each age group for the ICD9-CM coded hospitalization data is shown in blue, and the corresponding proportions for the fitted model are shown in red; error bars show the 95% credible intervals.

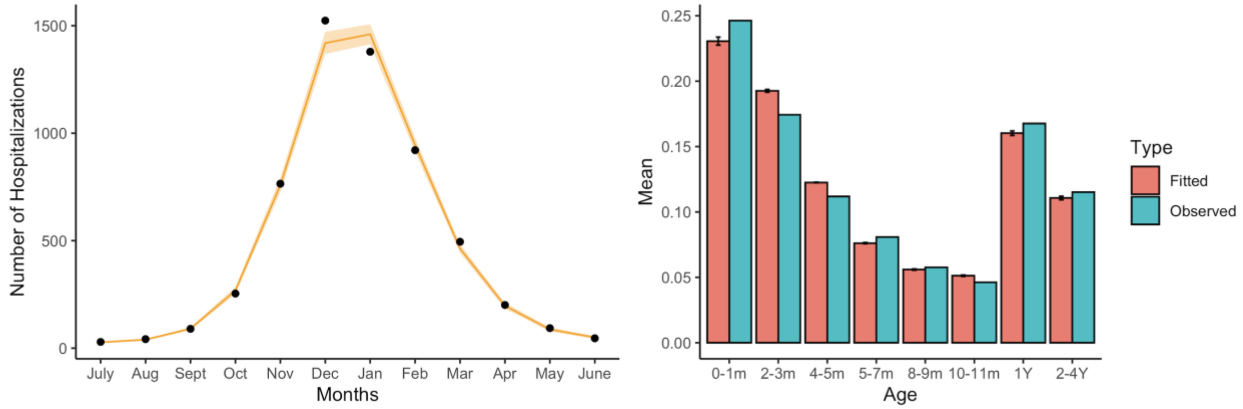

Supplementary Figure 2. Model fit to mean number of RSV hospitalization per month and age distribution in New York. On the left panel, the ICD9-CM coded hospitalization data is represented by the dots. The median of the fitted model is shown in solid yellow line while the shaded area around the line shows the 95% credible interval. On the right panel, the proportion of hospitalizations in each age group for the ICD9-CM coded hospitalization data is shown in blue, and the corresponding proportions for the fitted model are shown in red; error bars show the 95% credible intervals.

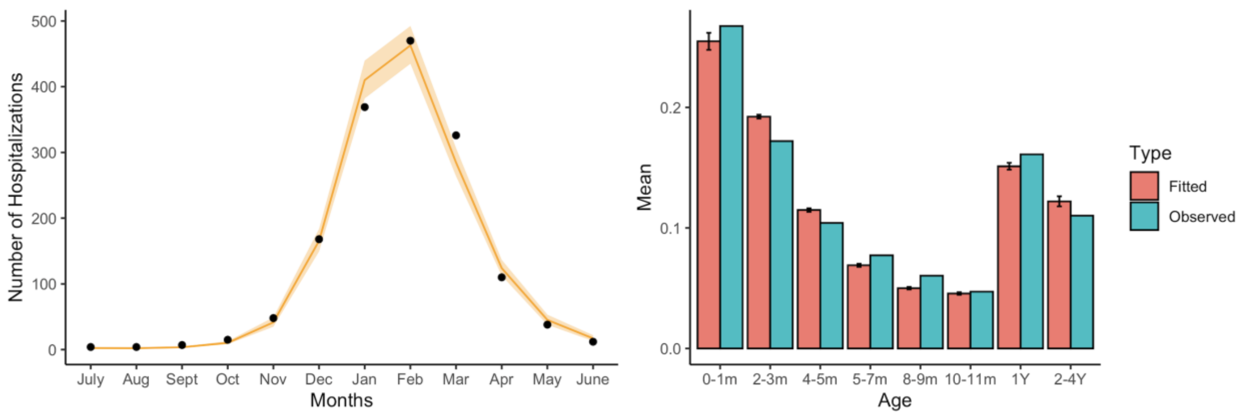

Supplementary Figure 3. Model fit to mean number of RSV hospitalization per month and age distribution in Washington. On the left panel, the ICD9-CM coded hospitalization data is represented by the dots. The median of the fitted model is shown in solid yellow line while the shaded area around the line shows the 95% credible interval. On the right panel, the proportion of hospitalizations in each age group for the ICD9-CM coded hospitalization data is shown in blue, and the corresponding proportions for the fitted model are shown in red; error bars show the 95% credible intervals.

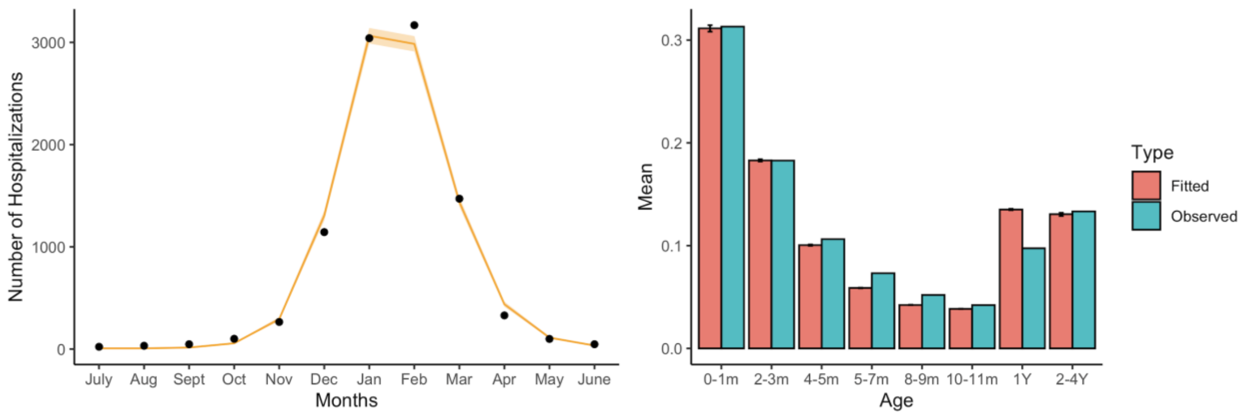

Supplementary Figure 4. Model fit to mean number of RSV hospitalization per month and age distribution in California. On the left panel, the ICD9-CM coded hospitalization data is represented by the dots. The median of the fitted model is shown in solid yellow line while the shaded area around the line shows the 95% credible interval. On the right panel, the proportion of hospitalizations in each age group for the ICD9-CM coded hospitalization data is shown in blue, and the corresponding proportions for the fitted model are shown in red; error bars show the 95% credible intervals.

Supplementary Table 1. State-specific estimated (median and 95% credible interval) transmission dynamic model parameters.

	New Jersey	New York	Washington	California
Basic reproductive number* (R_0)	10.37 (10.24, 10.52)	10.16 (10.12, 10.26)	10.11 (9.96, 10.26)	9.76 (9.72, 10.04)
Timing of seasonality (ϕ)	1.35 (1.34, 1.37)	1.36 (1.35, 1.37)	1.44 (1.43, 1.45)	1. 40 (1.40, 1.41)
Amplitude of seasonality (b_1)	0.20 (0.19, 0.21)	0.16 (0.16, 0.17)	0.20 (0.18, 0.21)	0.25 (0.23, 0.26)
Duration of maternal immunity and cocooning effects ($1/\omega*30.44$, in days)	90.84 (78.34, 104.43)	74.44 (69.02, 80.71)	41.07 (33.02, 50.70)	4.62 (3.41, 17.31)
Reporting fraction	0.76 (0.68, 0.87)	0.95 (0.84, 0.99)	0.63 (0.53, 0.81)	0.89 (0.62, 0.97)

*The basic reproductive number (R_0) was estimated from $R_0 = \frac{\det(\beta_{a,k})}{\gamma_1} = \frac{\det(qC_{a,k})}{\gamma_1}$, using the next-generation matrix method; the parameter q was fitted to the data, $C_{a,k}$ is the contact matrix scaled by the proportion of the population within each age class.

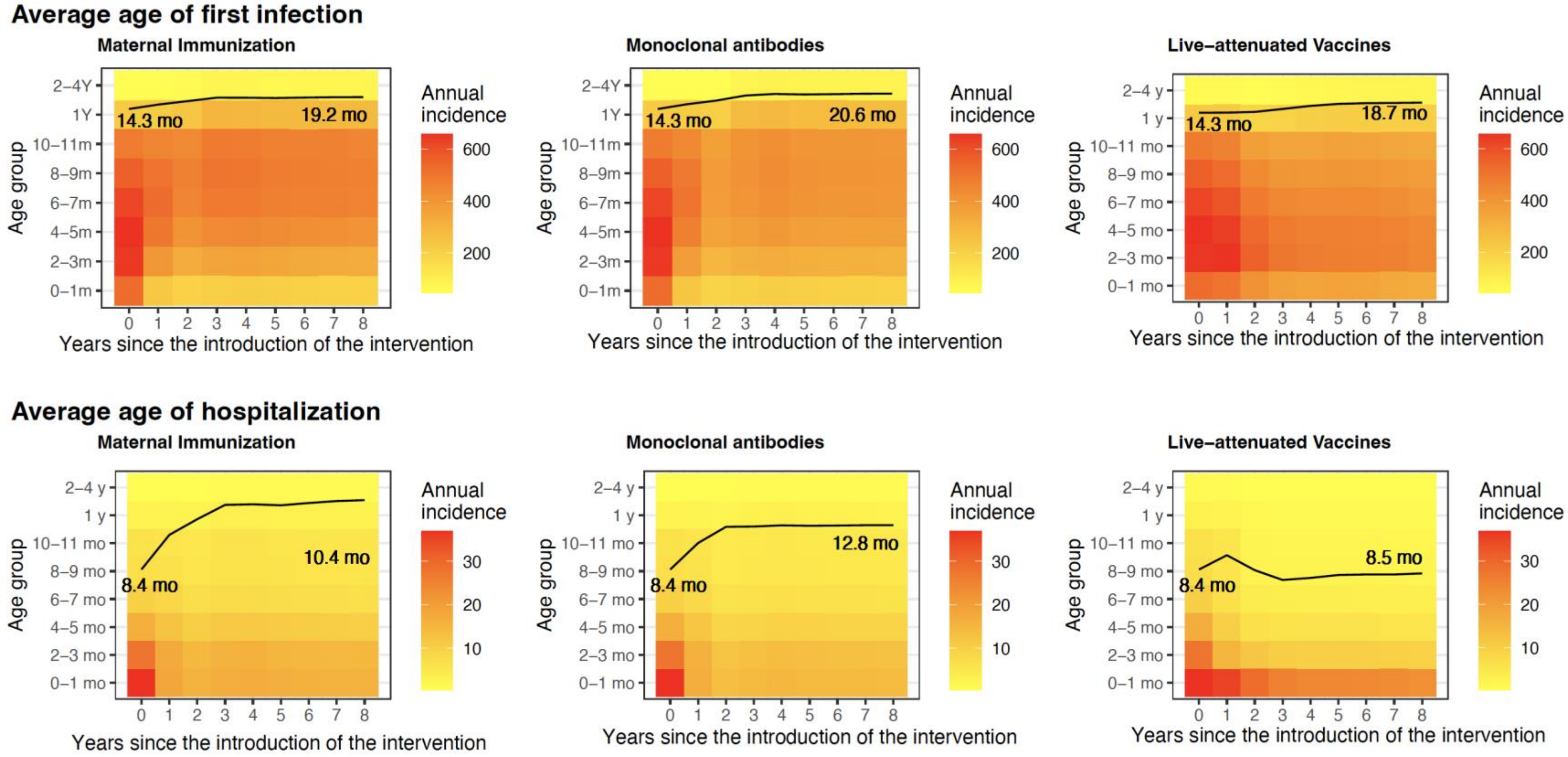

Supplementary Figure 5. Average age of first RSV infection and hospitalization before and after introduction of three RSV prevention strategies. The background color represents the incidence of RSV hospitalization per 1 000 people per year in each age group (y-axis) before and after vaccine introduction (time in years on the x-axis). Darker red colors indicate a higher incidence. The

black line and values indicate the mean age of first infection and hospitalization (in months) varies before and after the introduction of RSV prevention strategies.

Supplementary Table 2. Relative efficiency of Sep-Mar seasonal prevention strategy compared with year-round prevention strategy. The ratio of per-dose effectiveness between a seasonal program and a year-round program in each age group is shown for each strategy and age group. A ratio above 1 suggests that the seasonal program is more efficient.

Vaccination Strategy	Maternal immunization	Monoclonal antibodies	Live-attenuated vaccines
Age group			
**0-1 month**	1.45	1.37	0.92
**2-3 months**	1.19	1.10	1.29
**4-5 months**	0.91	0.86	1.08
**6-7 months**	0.67	0.70	0.94
**8-9 months**	0.36	0.64	0.90
**10-11 months**	-2.43	0.55	0.94
**Overall**	1.28	1.18	1.05

Sensitivity analysis

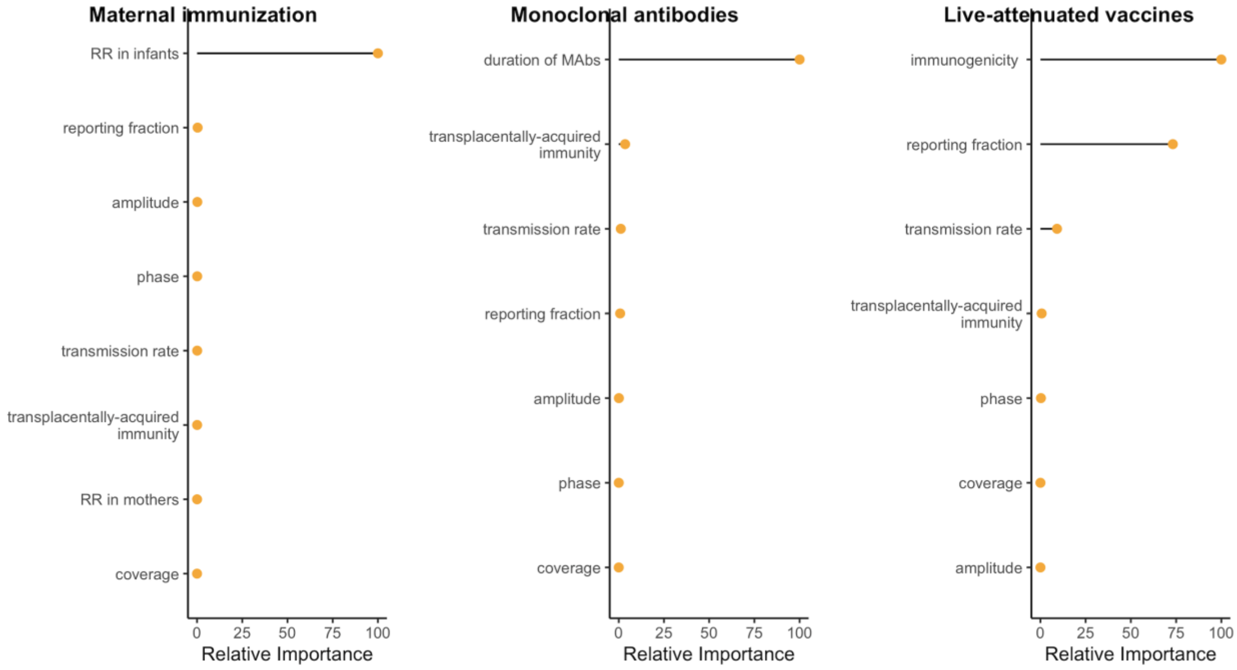

Supplementary Figure 6. Relative importance of variables for the predicted per-dose effectiveness of the three RSV prevention strategies. The relative importance of variables for model predictions of the per-dose effectiveness of (A) maternal immunization, (B) extended half-life monoclonal antibodies, and (C) live-attenuated vaccines are plotted. From top to bottom shows the most important factor in determining vaccine effectiveness to the least important factor in each prevention strategy.

Maternal immunization

To test how model structural assumptions may affect the overall effectiveness estimates of maternal immunization programs, we modified the model structure by assuming maternal immunization provides prolonged passive immunity to newborn infants while also lowering the risk of infection in mothers. We assumed the passive immunity from maternal immunization has an average duration of $1/\ \omega_{\mathrm{mat}} =$ 175 days (95% CI 150-200 days) in newborn infants.

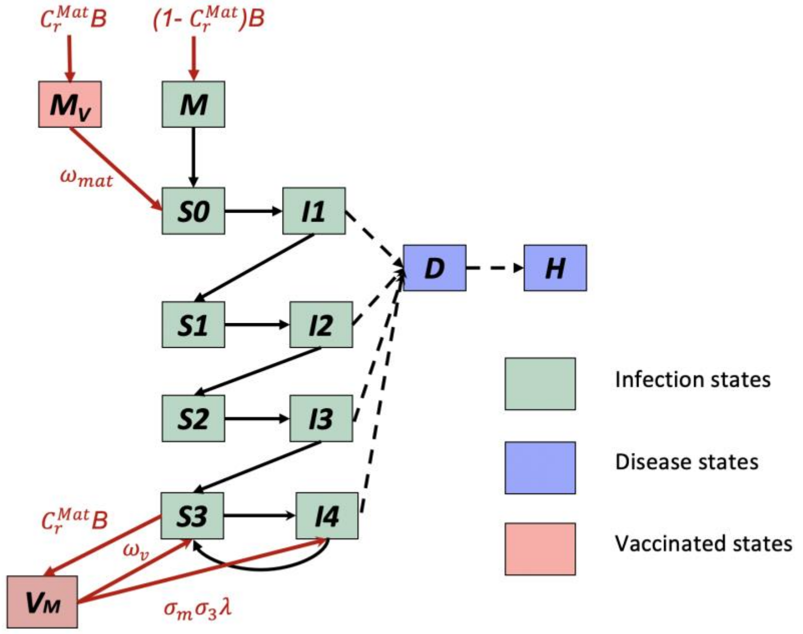

Supplementary Figure 7. Alternative model structure of maternal immunization programs. The green compartments represent RSV transmission dynamics. The purple compartments are the observational level diseases states. The pink compartments are immunized states.

We also tested two other assumptions to see how they affect the estimates of effectiveness, including (1) a shorter duration of vaccine-induced protection that lasts for 90 days and (2) a lower risk of infection in vaccinated mothers compared with unvaccinated mothers that ranges from 0.4 to 0.6.

Supplementary Table 3. Comparison of maternal immunization effectiveness under different assumptions. Overall effectiveness estimates for the original analysis and the sensitivity analyses. Medians and 95% prediction intervals are displayed.

Vaccination Strategy	Original maternal immunization (%)	Alternative model structure (%)	Shorter duration of vaccine-induced immunity (%)	Lower risk of infection in vaccinated mothers (%)
**0-1 month**	53 (34, 63)	46 (27, 69)	45 (29, 53)	54 (36, 64)
**2-3 months**	40 (25, 49)	37 (21, 53)	29 (19, 37)	41 (27, 50)
**4-5 months**	28 (17, 36)	29 (16, 39)	18 (10, 25)	29 (19, 39)
**6-7 months**	19 (11, 25)	21 (11, 28)	10 (4, 15)	20 (12, 28)
**8-9 months**	11 (6, 16)	13 (7, 19)	3 (0, 6)	12 (6, 18)
**10-11 months**	4 (0, 8)	7 (2, 12)	-3 (-5, 0)	4 (1, 9)
**1 Yr**	-12 (-25, -4)	-8 (-15, -4)	-12 (-24, -7)	-14 (-31, -8)
**2-4 Yrs**	-23 (-56, -7)	-23 (-42, -11)	-18 (-41, -8)	-32 (-73, -14)
**<5 Yrs**	24 (15, 30)	22 (13, 30)	18 (12, 23)	25 (16, 31)

An alternative model structure of maternal immunization yielded similar effectiveness estimates. Shorter duration of maternal immunity leads to a lower vaccine effectiveness, which is an 18% (12%, 23%) reduction in RSV-associated hospitalizations in children under 5 years old (compared with a 24% reduction if the duration is 5 months). Lower risk of RSV infection in vaccinated mothers yielded a marginal increase in effectiveness estimates in children under 5 years of age.

Live-attenuated vaccines

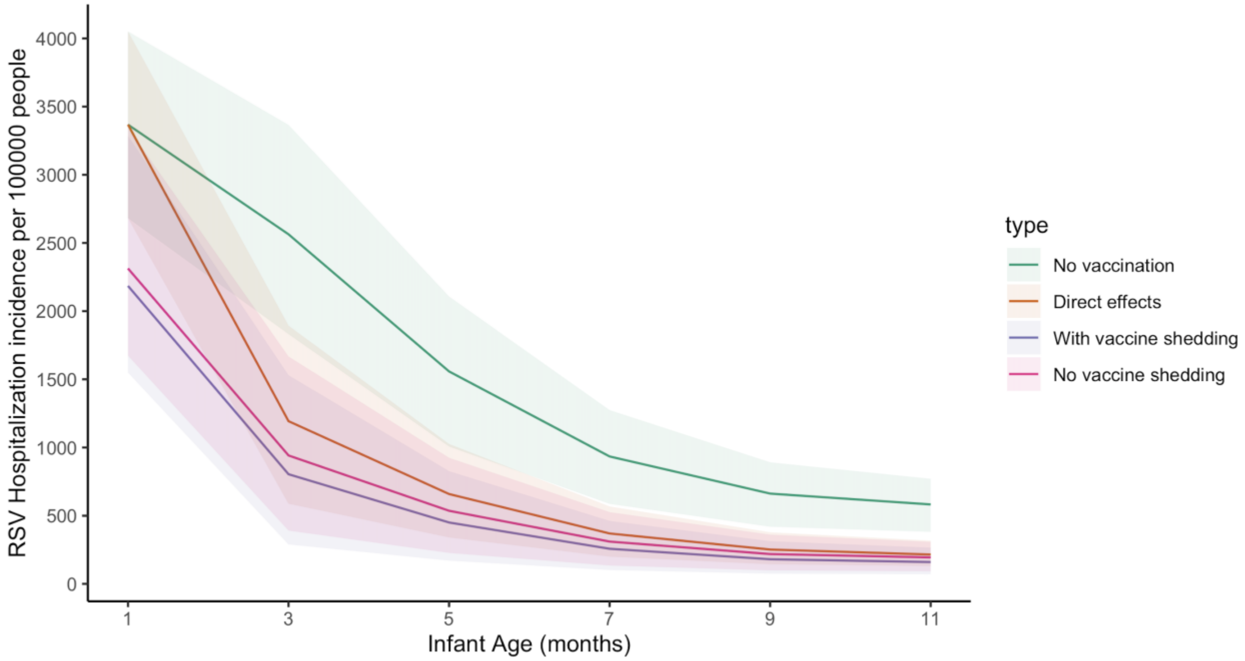

Supplementary Figure 8. Overall effects of live-attenuated vaccine with and without vaccine shedding. The green line shows the mean RSV hospitalization incidence by age (on the x-axis) assuming no vaccination. The orange line shows the model-predicted RSV hospitalization incidence by age in Year 7 following vaccine introduction accounting only for the model-predicted direct effects of live-attenuated vaccine (i.e., assuming no reduction in RSV transmission). The purple line shows the model-predicted RSV hospitalization incidence accounting for the overall effects of live-attenuated vaccine with vaccine shedding, while the pink line shows the overall effects without vaccine shedding (i.e. assuming $\lambda_v = 0$). The color shadows show the 95% prediction intervals as indicated by the legend.

We tested the assumption of one booster dose of live-attenuated vaccines for infants aged 4-5 months. We assumed a booster dose would also induce both humoral and cellular immune responses comparable to an additional natural infection.

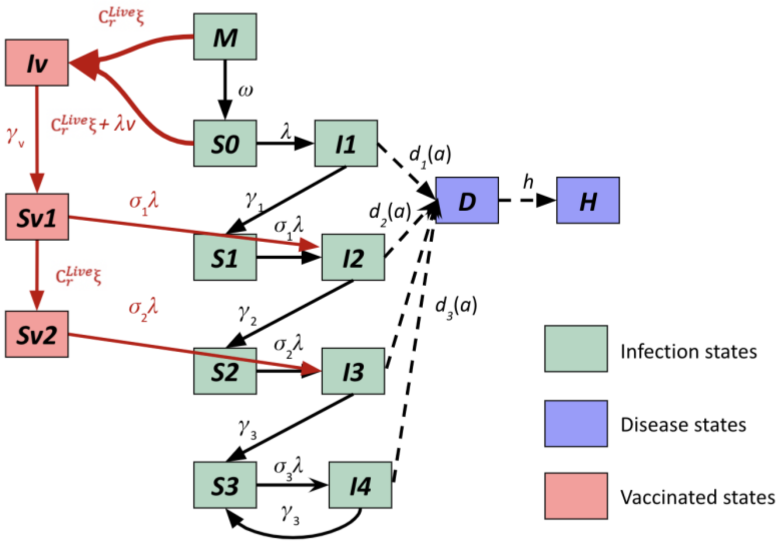

Supplementary Figure 9. Alternative model structure of a booster dose of live-attenuated vaccines for infants. The green compartments represent RSV transmission dynamics. The purple compartments are the observational level diseases states. The pink compartments are immunized states.

The booster dose of the live-attenuated RSV vaccine increased the overall vaccine effectiveness in every age group in children under 5 years of age. Infants aged 4-5 months benefited the most from the live-attenuated booster dose, resulting in an 83% (68%, 94%) reduction in RSV-associated hospitalizations (Supplementary Table 4). Overall, live-attenuated vaccines with a booster dose are predicted to lead to a 67% (49%, 80%) reduction in RSV-associated

hospitalizations in children under 5 years old (compared with a 53% reduction without a booster dose).

Supplementary Table 4. Comparison of effectiveness of live-attenuated vaccine with and without booster dose. Overall effectiveness estimates for the original analysis and the sensitivity analysis including a booster dose. Medians and 95% prediction intervals are displayed.

Vaccination Strategy	Live-attenuated vaccines without booster dose (%)	Live-attenuated vaccines with booster dose (%)
**0-1 month**	31 (11, 45)	44 (14, 61)
**2-3 months**	64 (44, 79)	71 (46, 85)
**4-5 months**	65 (50, 77)	83 (68, 94)
**6-7 months**	66 (53, 77)	83 (68, 94)
**8-9 months**	66 (54, 76)	83 (68, 93)
**10-11 months**	65 (54, 76)	82 (68, 93)
**1 Yr**	61 (51, 72)	78 (54, 90)
**2-4 Yrs**	50 (39, 63)	66 (50, 82)
**<5 Yrs**	53 (39, 64)	67 (49, 80)

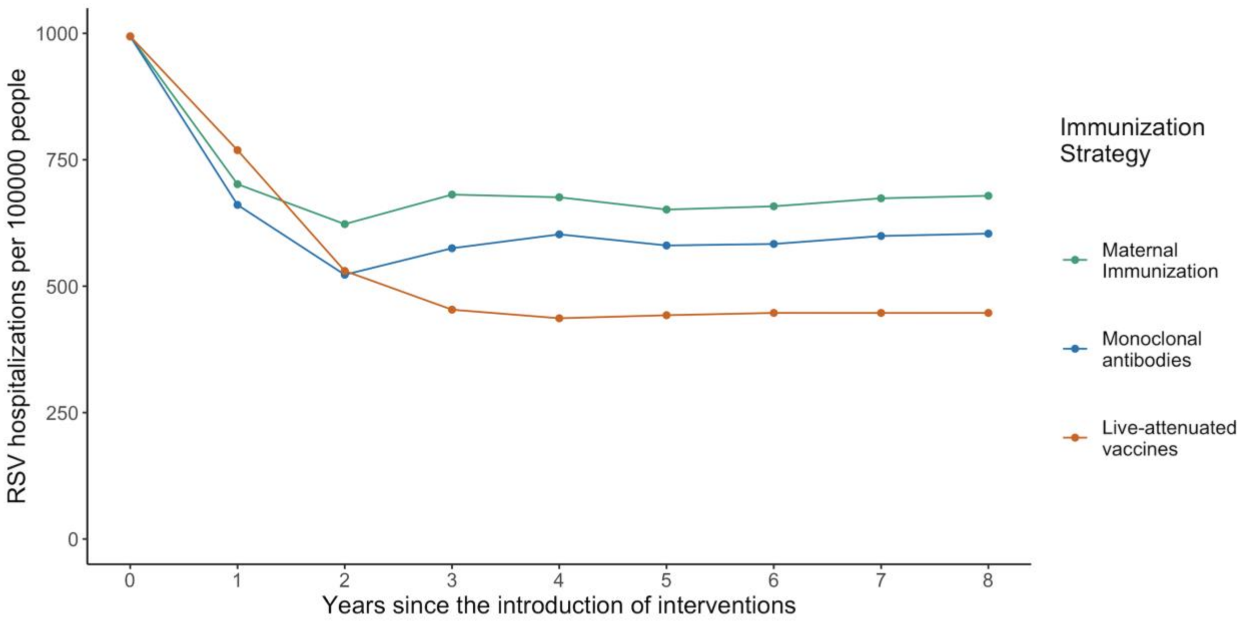

Supplementary Figure 10. Annual RSV hospitalization incidence per 100,000 people for all children <2 years of age before and after the introduction of three RSV prevention strategies. Year 0 corresponds to the RSV hospitalization incidence before the introduction of RSV prevention strategies. The predicted RSV hospitalization incidence over time is plotted for Years 1 to 8 after the introduction of the intervention. We assumed 85%-95% coverage for all three interventions. The color of the lines corresponds to the different RSV prevention strategies as indicated by the legend.

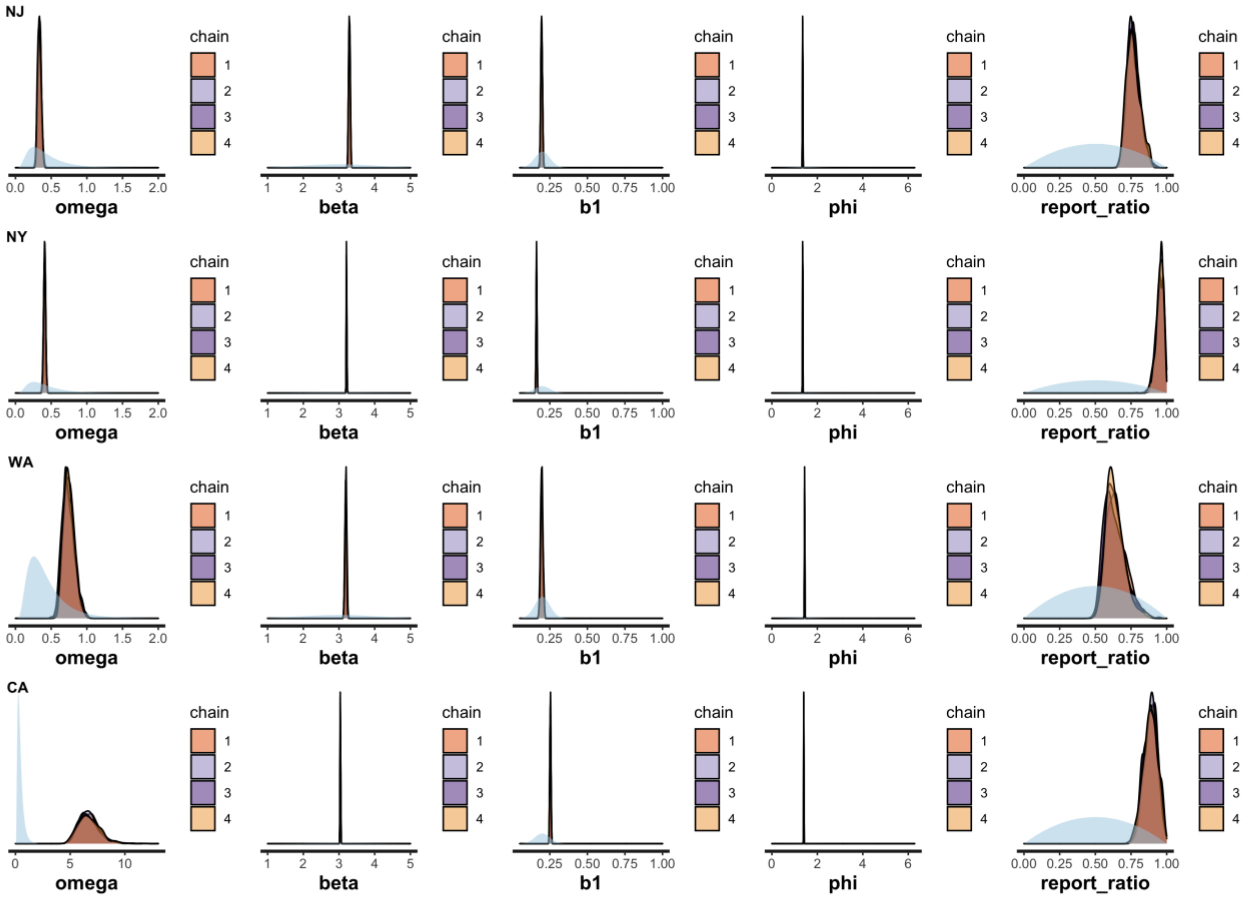

Supplementary Figure 11. Prior and posterior distributions of state-specific estimated transmission dynamic model parameters. The light blue areas represent the weakly-informative prior distribution for each parameter. The color shaded areas represent the posterior distribution of state-specific estimated transmission dynamic model parameters in each chain.

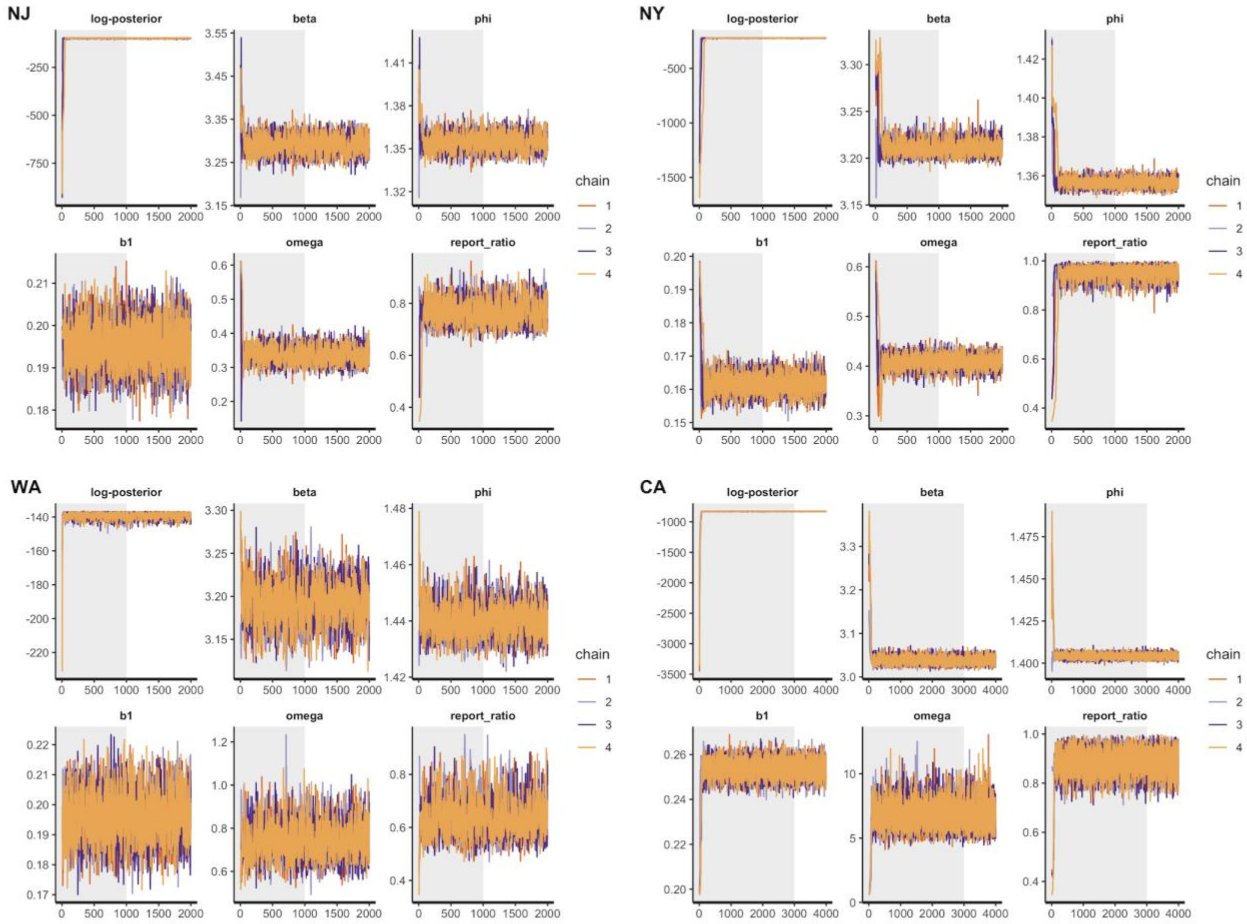

Supplementary Figure 12. Parameter trace plots for four states. Posterior draws of the log posterior model probability and five parameters from New Jersey (NJ), New York (NY), Washington (WA), and California (CA). The grey shaded areas indicate the burn-in period. The color lines indicate four chains of posterior draws. All parameters converged well in all states.

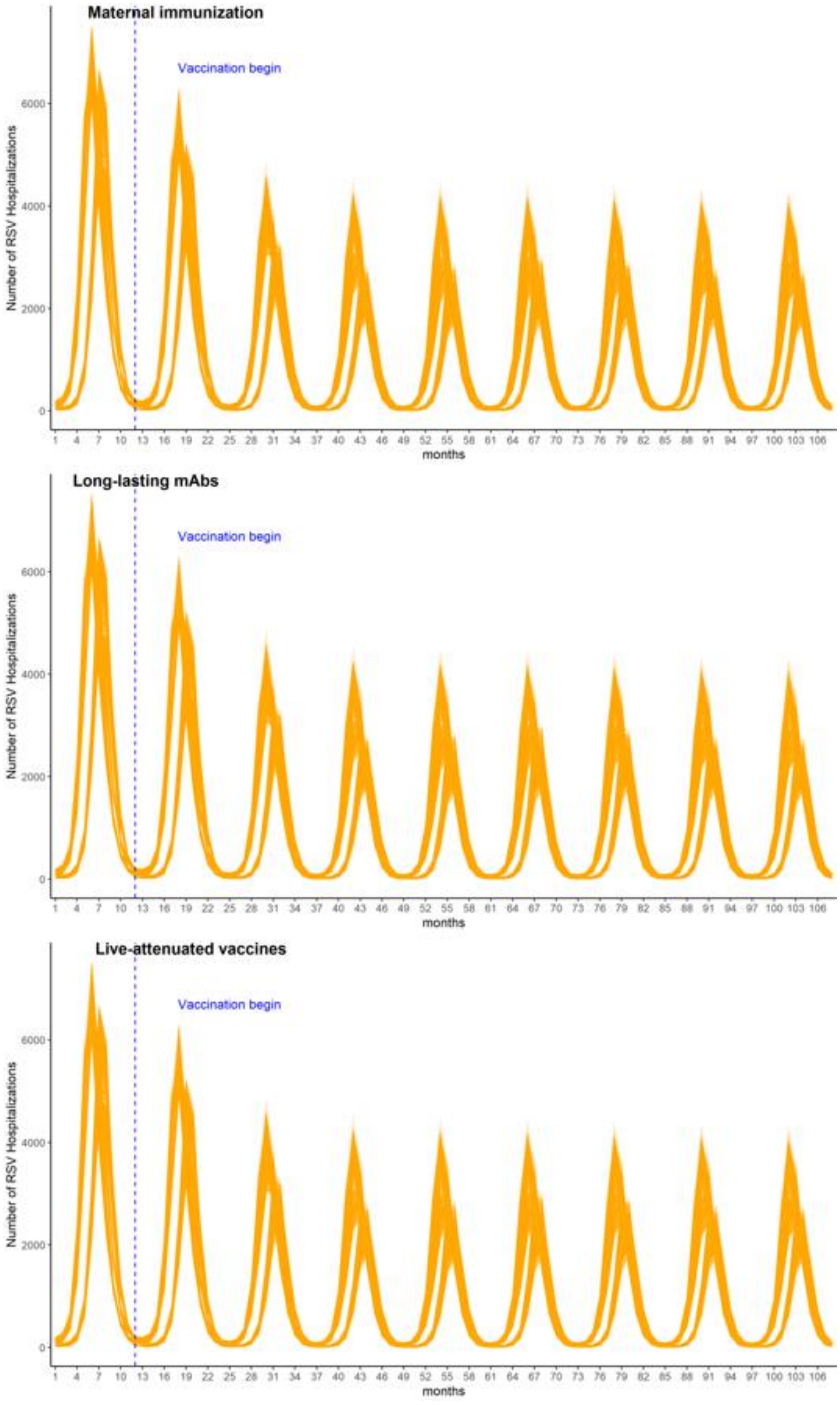

Maternal immunization
Vaccination begin
Number of RSV Hospitalizations
0
2000
4000
6000
1 4 7 10 13 16 19 22 25 28 31 34 37 40 43 46 49 52 55 58 61 64 67 70 73 76 79 82 85 88 91 94 97 100 103 106
months
Long-lasting mAbs
Vaccination begin
Number of RSV Hospitalizations
0
2000
4000
6000
1 4 7 10 13 16 19 22 25 28 31 34 37 40 43 46 49 52 55 58 61 64 67 70 73 76 79 82 85 88 91 94 97 100 103 106
months
Live-attenuated vaccines
Vaccination begin
Number of RSV Hospitalizations
0
2000
4000
6000
1 4 7 10 13 16 19 22 25 28 31 34 37 40 43 46 49 52 55 58 61 64 67 70 73 76 79 82 85 88 91 94 97 100 103 106
months

Supplementary Figure 13. RSV-associated hospitalizations from each model run. Total number of RSV hospitalizations over time in months (on the x-axis) for (A) the maternal immunization strategy, (B) extended half-life monoclonal antibodies, and (C) live-attenuated vaccines. Each orange line represents the result of one set of parameter combinations. The dotted blue line at month 12 represents the time when the intervention is introduced.

Supplementary References:

1. Prem K, Cook AR, Jit M: **Projecting social contact matrices in 152 countries using contact surveys and demographic data**. *PLoS Comput Biol* 2017, **13**(9):e1005697.
2. Wallinga J, Teunis P, Kretzschmar M: **Using data on social contacts to estimate age-specific transmission parameters for respiratory-spread infectious agents**. *American journal of epidemiology* 2006, **164**(10):936-944.
