# Supplementary material for: Predicted effectiveness of vaccines and extended half-life monoclonal antibodies against RSV hospitalizations in children
Source: NPJ Vaccines. 2022 Oct 27;7:127. doi: 10.1038/s41541-022-00550-5 (PMC9612629; doi:10.1038/s41541-022-00550-5)
Supplement: Supplementary file 2 — REPORTING SUMMARY [file 41541_2022_550_MOESM2_ESM.pdf]

nature portfolio

Corresponding author(s): Zhe Zheng

Last updated by author(s): Aug 19, 2022

Reporting Summary

Nature Portfolio wishes to improve the reproducibility of the work that we publish. This form provides structure for consistency and transparency in reporting. For further information on Nature Portfolio policies, see our Editorial Policies and the Editorial Policy Checklist.

Statistics

For all statistical analyses, confirm that the following items are present in the figure legend, table legend, main text, or Methods section.

n/a	Confirmed	
☒	☐	The exact sample size (*n*) for each experimental group/condition, given as a discrete number and unit of measurement
☒	☐	A statement on whether measurements were taken from distinct samples or whether the same sample was measured repeatedly
☒	☐	The statistical test(s) used AND whether they are one- or two-sided<br>*Only common tests should be described solely by name; describe more complex techniques in the Methods section.*
☒	☐	A description of all covariates tested
☒	☐	A description of any assumptions or corrections, such as tests of normality and adjustment for multiple comparisons
☐	☒	A full description of the statistical parameters including central tendency (e.g. means) or other basic estimates (e.g. regression coefficient) AND variation (e.g. standard deviation) or associated estimates of uncertainty (e.g. confidence intervals)
☒	☐	For null hypothesis testing, the test statistic (e.g. *F*, *t*, *r*) with confidence intervals, effect sizes, degrees of freedom and *P* value noted<br>*Give P values as exact values whenever suitable.*
☐	☒	For Bayesian analysis, information on the choice of priors and Markov chain Monte Carlo settings
☒	☐	For hierarchical and complex designs, identification of the appropriate level for tests and full reporting of outcomes
☒	☐	Estimates of effect sizes (e.g. Cohen's *d*, Pearson's *r*), indicating how they were calculated

Our web collection on statistics for biologists contains articles on many of the points above.

Software and code

Policy information about availability of computer code

Data collection	Individual-level hospital discharge data were obtained from the State Inpatient Databases of the Healthcare Cost and Utilization Project, maintained by the Agency for Healthcare Research and Quality (purchased through the HCUP Central Distributor). No code or software for data collection was used.
Data analysis	We performed Bayesian inference with STAN, (a gradient-based sampling technique) in R version 4.0.2. All other analyses were performed using R version 4.0.2 as well.

For manuscripts utilizing custom algorithms or software that are central to the research but not yet described in published literature, software must be made available to editors and reviewers. We strongly encourage code deposition in a community repository (e.g. GitHub). See the Nature Portfolio guidelines for submitting code & software for further information.

Data

Policy information about availability of data

All manuscripts must include a data availability statement. This statement should provide the following information, where applicable:

- Accession codes, unique identifiers, or web links for publicly available datasets
- A description of any restrictions on data availability
- For clinical datasets or third party data, please ensure that the statement adheres to our policy

The data and code that support the findings of this study are available via GitHub (https://github.com/weinbergerlab/rsv-transmission-model). The hospitalization data are not available publicly but can be obtained upon signing a data use agreement with the Agency for Healthcare Research and Quality.

Human research participants

Policy information about studies involving human research participants and Sex and Gender in Research.

Reporting on sex and gender	This study used de-identified patient data. The datasets used in this study contain all inpatient care records in participating states. This study did not involve recruitment of human research participants.
Population characteristics	This study used de-identified patient data. The datasets used in this study contain all inpatient care records in participating states. The data covered July 2005 to June 2014 for three states (New York, New Jersey, Washington) and from July 2003 to June 2011 for California.
Recruitment	This study did not involve recruitment of human research participants
Ethics oversight	Ethics committee/IRB of Yale University waived ethical approval for this work

Note that full information on the approval of the study protocol must also be provided in the manuscript.

Field-specific reporting

Please select the one below that is the best fit for your research. If you are not sure, read the appropriate sections before making your selection.

☐ Life sciences ☐ Behavioural & social sciences ☒ Ecological, evolutionary & environmental sciences

For a reference copy of the document with all sections, see nature.com/documents/nr-reporting-summary-flat.pdf

Ecological, evolutionary & environmental sciences study design

All studies must disclose on these points even when the disclosure is negative.

Study description	We used age-structured transmission models to evaluate the possible impact of various RSV prevention strategies including maternal immunization, live-attenuated vaccines, and long-lasting mAbs.
Research sample	The datasets used in this study contain all inpatient care records in participating states. The data covered July 2005 to June 2014 for three states (New York, New Jersey, Washington) and from July 2003 to June 2011 for California.
Sampling strategy	No sampling was done to obtain any subset of inpatient care records.
Data collection	Individual-level hospital discharge data were obtained from the State Inpatient Databases of the Healthcare Cost and Utilization Project, maintained by the Agency for Healthcare Research and Quality (purchased through the HCUP Central Distributor).
Timing and spatial scale	The data covered July 2005 to June 2014 for three states (New York, New Jersey, Washington) and from July 2003 to June 2011 for California.
Data exclusions	NA
Reproducibility	The data and code that support the findings of this study are available via GitHub (https://github.com/weinbergerlab/rsv-transmission-model). The hospitalization data are not available publicly but can be obtained upon signing a data use agreement with the Agency for Healthcare Research and Quality.
Randomization	NA
Blinding	NA

Did the study involve field work? ☐ Yes ☒ No

Reporting for specific materials, systems and methods

We require information from authors about some types of materials, experimental systems and methods used in many studies. Here, indicate whether each material, system or method listed is relevant to your study. If you are not sure if a list item applies to your research, read the appropriate section before selecting a response.

Materials & experimental systems

n/a	Involved in the study
☒	☐ Antibodies
☒	☐ Eukaryotic cell lines
☒	☐ Palaeontology and archaeology
☒	☐ Animals and other organisms
☒	☐ Clinical data
☒	☐ Dual use research of concern

Methods

n/a	Involved in the study
☒	☐ ChIP-seq
☒	☐ Flow cytometry
☒	☐ MRI-based neuroimaging
